# Developing prediction models for symptom severity around the time of discharge from a tertiary-care program for treatment-resistant psychosis

**DOI:** 10.3389/fpsyt.2023.1181740

**Published:** 2023-06-07

**Authors:** Lik Hang N. Lee, Ric M. Procyshyn, Randall F. White, Kristina M. Gicas, William G. Honer, Alasdair M. Barr

**Affiliations:** ^1^Faculty of Medicine, University of Ottawa, Ottawa, ON, Canada; ^2^Department of Psychiatry, University of British Columbia, Vancouver, BC, Canada; ^3^British Columbia Mental Health and Substance Use Services Research Institute, Vancouver, BC, Canada; ^4^Department of Psychology, York University, Toronto, ON, Canada; ^5^Department of Anesthesiology, Pharmacology & Therapeutics, University of British Columbia, Vancouver, BC, Canada

**Keywords:** schizophrenia, psychosis, prediction model, tertiary care, treatment resistance

## Abstract

Antipsychotics are the only therapeutic class indicated in the symptomatic management of psychotic disorders. However, individuals diagnosed with schizophrenia or schizoaffective disorder may not always benefit from these first-line agents. This refractoriness to conventional treatment can be difficult to address in most clinical settings. Therefore, a referral to a tertiary-care program that is better able to deliver specialized care in excess of the needs of most individuals may be necessary. The average outcome following a period of treatment at these programs tends to be one of improvement. Nonetheless, accurate prognostication of individual-level responses may be useful in identifying those who are unlikely to improve despite receiving specialized care. Thus, the main objective of this study was to predict symptom severity around the time of discharge from the Refractory Psychosis Program in British Columbia, Canada using only clinicodemographic information and prescription drug data available at the time of admission. To this end, a different boosted beta regression model was trained to predict the total score on each of the five factors of the Positive and Negative Syndrome Scale (PANSS) using a data set composed of 320 hospital admissions. Internal validation of these prediction models was then accomplished by nested cross-validation. Insofar as it is possible to make comparisons of model performance across different outcomes, the correlation between predictions and observations tended to be higher for the negative and disorganized factors than the positive, excited, and depressed factors on internal validation. Past scores had the greatest effect on the prediction of future scores across all 5 factors. The results of this study serve as a proof of concept for the prediction of symptom severity using this specific approach.

## Introduction

Schizophrenia is a neurodevelopmental disorder characterized by altered brain connectivity and synaptic dysregulation (1–5). Antipsychotics are the primary therapeutic class indicated in the symptomatic management of psychotic disorders such as schizophrenia. Unfortunately, while most individuals show some therapeutic response to these drugs, an inadequate response to this first-line treatment is observed in approximately one-quarter of patients with first-episode psychosis (6–10). Moreover, there also exists a subset of patients who become refractory to treatment following a variable period of response (11). Consequently, specialized approaches, like the timely initiation of the atypical antipsychotic clozapine (12) may be warranted if there is an ongoing lack of clinical improvement despite multiple antipsychotic trials of adequate dose and duration.

Tertiary-care programs have shown promise in the effective delivery of these approaches under real world conditions (13–16). In a retrospective study investigating antipsychotic prescribing patterns at a tertiary-care program for treatment-resistant psychosis, we observed fewer instances of antipsychotic polypharmacy and a greater use of clozapine when comparing the drugs prescribed at discharge to those prescribed at admission (17). This trend towards a more evidence-based approach to pharmacotherapy coincided with decreases in symptom severity as quantified using the original three-factor Positive and Negative Syndrome Scale (PANSS) (18). Nevertheless, some individuals discharged from the program remained without clinical improvement. Given both the dearth of prognostic models in this patient population and the potential utility in identifying non-responders to specialized treatment approaches as early into their admission as possible, it was of interest to determine if symptom severity around the time of discharge can be predicted using only the information available at, or shortly after the time of admission, to a tertiary-care program. To this end, the current study aimed to develop and internally validate models for the prediction of PANSS scores around the time of discharge following a re-scoring of values to more accurately reflect the multidimensional symptomatology of treatment-resistant psychosis (19). An exploratory data analysis was then performed to identify predictors routinely selected by the model fitting procedure for further study in the future.

## Materials and methods

The clinical research ethics board at the University of British Columbia approved the study protocol and granted a waiver of consent for the secondary use of retrospective chart review data, as previously (20, 21). Data processing, statistical analysis, and data visualization were accomplished using R (v. 3.5.1). Add-on packages facilitating these tasks include tidyverse (22), gamlss (23), gamboostLSS (24), rsample (25), ggplot2 (26), viridis (27), cowplot (28), and extrafont (29).

### Overview of the model fitting procedure

A component-wise gradient boosting approach to beta regression (30, 31) was used to predict symptom severity around the time of discharge from a tertiary-care program for treatment-resistant psychosis. This involved the training of five distinct models, one for each symptom domain routinely identified by previous factor analyses of PANSS scores (32).

This approach to predictive modelling has the following advantages. If *n* is the number of items included in a given factor of the PANSS, then the total score on that factor can only take on integer values in the interval [*n*,7*n*]. These lower and upper bounds limit the utility of linear regression models (33), but a rescaling of the scores as decimal proportions of the upper bound would enable their prediction by beta regression models instead. These rescaled values would normally need to be continuous (34), but discrete proportions can be predicted in the same way if they reflect an underlying continuous beta distribution (35). We consider this to be a tenable assumption because the routine practice of reporting total scores only makes sense if they approximate the latent variables of interest. Furthermore, while maximum likelihood can be used to estimate parameters in beta regression, component-wise gradient boosting offers two distinct advantages. First, the functional form of non-linear relationships do not need to be specified beforehand if they are modelled using penalized regression spline (P-spline) base learners (36). Second, early stopping of the boosting algorithm can result in both shrinkage and variable selection since the additive predictor (*η*) is only updated with a fixed proportion of the fit of the base learner that best fits the negative gradient vector at each iteration (24). These are both desirable properties given how little is known about predictors of symptom severity in treatment-resistant psychosis following hospitalization at a tertiary care program.

Working within the framework of generalized additive models for location, scale and shape (23), the location ( μ ) and scale ( σ ) parameters of each beta distribution were related to their respective additive predictors ( $\eta_{\mu }$ and $\eta_{\sigma }$ ) by a logit link function. The ensuing model was then trained using a non-cyclic algorithm capable of updating only one of these additive predictors in each iteration (37). Specifically, the additive predictor that most improved the empirical risk of the model following the inclusion of its best fitting base learner was updated with one-tenth of this fit. This intentional slowing of the learning rate enabled both shrinkage estimation and variable selection to occur should the algorithm be allowed to come to an early stop (24).

The optimal number of iterations with which to update both additive predictors in a specific model was identified by searching a vector of values for the number of iterations that minimizes the average predictive risk in 10-fold cross-validation. This hyperparameter, hereafter referred to as *m*_*stop*_, was arbitrarily limited to a maximum of 1,000 iterations should earlier stopping not occur. Once the model has been updated to *m*_*stop*_ iterations, the predicted values of the distribution parameters were used to estimate the mean and the variance of the beta distribution based on the relationships: E(X)=μ ; $\mathrm{Var}\left(X\right)=\mu \left(1-\mu \right)\sigma^{2}$ .

Nested cross-validation was then used in the absence of an independent testing data set to internally validate this model fitting procedure (38, 39). Model selection (i.e., the tuning of $m_{\mathrm{stop}}$ ) was accomplished in the inner loop by 10-fold cross-validation. Model assessment (i.e., the estimation of prediction error) was accomplished in the outer loop by 100 times 10-fold cross-validation. In essence, the model fitting procedure was repeated 1,000 times, but instead of using all of the data to train each model, a different nine-tenths of the data was selected as the training data set in each iteration. This freed up the remaining one-tenth of the data to be used as a testing data set for the model in question.

### Study population

Data were retrospectively abstracted from the charts of individuals admitted to the Refractory Psychosis Program at Riverview Hospital in Coquitlam, British Columbia, Canada for psychosis not improved by conventional treatment despite multiple antipsychotic trials of adequate dose and duration as documented by the referring physician (17). Those without antipsychotic use at admission or discharge, PANSS scores around the time of admission or discharge, or a primary diagnosis of schizophrenia or schizoaffective disorder based on the criteria outlined in either the Diagnostic and Statistical Manual III-R or IV were excluded upon identification. If an individual had multiple admissions to this program over the observation period, then data from only the most recent admission were included in the data set because later admissions were hypothesized to reflect higher degrees of treatment resistance. Following listwise deletion of admissions with missing data, 320 of 325 hospitalizations meeting the above selection criteria remained in the data set.

### Variables

No actions were taken to blind the assessment of predictors for the outcome. While information routinely available around the time of admission had already been gathered, data for which the veracity was dependent on the reliability of the historian were excluded owing to the absence or inaccessibility of collateral information. The list of candidate predictors was therefore solely derived from the following set of variables: sex, age, diagnosis, year of admission, history of a prior clozapine trial, medications, and PANSS scores. A more detailed discussion of these variables is available elsewhere (17).

Sex, psychiatric diagnosis, and history of a prior clozapine trial were treated as binary data. Age and year of admission were treated as numeric data precise to one decimal place. The list of regularly prescribed medications (i.e., those not prescribed *pro re nata*) were initially processed by sorting the individual drugs into their respective therapeutic classes. Antipsychotic utilization was then quantified by the prescribed daily dose (PDD) to defined daily dose (DDD) ratio (i.e., PDD:DDD) (40) as was done previously (17). Patterns of antipsychotic use were further characterized using two binary variables to separately denote the use of clozapine and antipsychotic polypharmacy at admission. Contrary to the approach taken for antipsychotics as a therapeutic class, the use of antidepressants, mood stabilizers, benzodiazepines, and anticholinergics was strictly limited to qualitative representation by way of binary variables since these were not always prescribed for their primary indication [i.e., antidepressants and mood stabilizers may instead be used as off-label adjunctive agents to augment antipsychotic response (41)]. All remaining therapeutic classes were excluded from further consideration as candidate predictors since these were used to treat comorbidities present in only a minor subset of the study population. Factor-specific PANSS scores were obtained by summing the scores of individual items that had been grouped together according to the consensus five-factor model developed by Wallwork et al. (32) to approximate symptom severity in a manner that better reflects the underlying symptom dimensions in psychotic disorders.

After dummy coding the binary data and mean-centering the numeric data, a linear base learner was specified for each candidate predictor to model its effect on the distribution parameter of interest. A P-spline base learner was also specified for each numeric predictor to entertain the possibility of non-linear effects. Both linear and P-spline base learners were assigned one degree of freedom to mitigate any undue preference for increasing model complexity (42).

### Outcomes

No actions were taken to blind the assessment of the different outcomes. Factor-specific PANSS scores around the time of discharge were derived in the same manner and for the same reason as those approximating symptom severity around the time of admission. However, the summed totals required further rescaling using the formula: $y_{\mathrm{rescaled}}=\left(y-n+1\right)/\left(7n-n+1\right)$ , where n is the number of PANSS items assigned to a particular factor, y is the total score on the original scale, and $y_{\mathrm{rescaled}}$ is an approximation of that score as a decimal proportion of 7n (i.e., the maximum score if all items in that factor were rated “extreme”) (35). No additional steps were necessary to constrain the rescaled values to the interval (0,1) as is required for beta regression since the numerator precludes values of 0 and the maximum score of 7n was not observed across any of the factors.

### Statistical analysis

Agreement between model predictions and their respective observations was quantified within the outer loop of the nested cross-validation by pseudo *R*^2^ (34, 43) and root mean squared error (RMSE). Differences between training and testing data sets in terms of these two metrics were interpreted as optimism in the apparent performance of boosted beta regression. A qualitative assessment was facilitated by the fitting of locally weighted least squares regression smoothers using the *loess* function in R. Probabilistic calibration was also assessed using probability integral transform (PIT) histograms (44, 45) following the discretization of predicted beta distributions (35). The five models trained on all data were not updated in light of the findings of this internal validation.

### Exploratory data analysis

Since the refitting of each model in the outer loop of the nested cross-validation involved training data sets that were largely composed of the same observations (i.e., eight-ninths of the data are shared between any two training data sets in a single 10-fold cross-validation), the set of candidate predictors selected by the non-cyclical algorithm was expected to share some similarities between runs and repeats. To this end, the proportion of refitted models in which the base learner or base learners specified for a given variable were used to update an additive predictor at least once before termination of the algorithm was quantified and reported as the variable selection frequency for that particular additive predictor.

A series of partial dependence plots were constructed from the refitted models trained in the outer loop of the nested cross-validation to examine the marginal effect of each variable on predicted values of the distribution parameters (46). A second set of partial dependence plots was constructed from the five models trained on the full data set following the discretization of predicted beta distributions to examine the marginal effect of each included variable on the probability of obtaining a specific score around the time of discharge.

## Results

The variables available at or around the time of admission that were given consideration as candidate predictors of symptom severity around the time of discharge are summarized in Table 1. Briefly, 58.1% of the 320 individuals admitted to the Refractory Psychosis Program for management of treatment-resistant psychosis had previously been treated with clozapine, but only 20.3% of the study population were prescribed clozapine at admission. This was comparable in rate to the prescription of oral anticholinergics for the prophylactic or symptomatic management of extrapyramidal symptoms (20.6%). Total antipsychotic utilization generally exceeded the defined daily dose for conventional management of psychotic disorders as evidenced by a median PDD:DDD of 1.81, but this tendency toward higher doses was observed even in the absence of antipsychotic polypharmacy in 48.1% of the study population. The concurrent prescription of other psychotropics was relatively common despite the selection criteria excluding individuals with treatment-resistant psychosis attributable to either depression or bipolar disorder. For example, antidepressants, though less frequently prescribed compared to mood stabilizers and benzodiazepines (21.9% versus 37.5 and 31.9%), were also similar in rate to clozapine at admission.

**Table 1:** Variables available at or around the time of admission to a tertiary care program for treatment-resistant psychosis given consideration as candidate predictors of symptom severity around the time of discharge.

Candidate predictors	Median [Q1, Q3] or Count (%)
Year of admission	2001.6 [1998.1, 2005.3]
Age	36.5 [27.0, 44.0]
Sex	
Male	190 (59.4%)
Female	130 (40.6%)
Psychotic disorder	
Schizophrenia	221 (69.1%)
Schizoaffective disorder	99 (30.9%)
Previous pharmacological interventions	
Prior clozapine trial	186 (58.1%)
Current pharmacological interventions	
Total antipsychotic utilization	1.81 [1.09, 2.75]
Clozapine	65 (20.3%)
Antipsychotic polypharmacy	166 (51.9%)
Antidepressant(s)	70 (21.9%)
Mood stabilizer(s)	120 (37.5%)
Benzodiazepine(s)	102 (31.9%)
Anticholinergic(s)	66 (20.6%)
Symptom severity by total PANSS scores	
Positive	16 [13, 19]
Negative	19 [16, 22.25]
Disorganized	11 [9, 13]
Excited	11 [8, 14]
Depressed	7 [5, 9]

*N* = 320. PANSS, Positive and Negative Syndrome Scale. Antipsychotic utilization was quantified by the prescribed daily dose (PDD) to defined daily dose (DDD) ratio (World Health Organization, 2020). PDD:DDD ratios were summed if antipsychotic polypharmacy was observed at admission. Drugs prescribed *pro re nata* have been excluded from the analysis. The theoretical range of total scores on each of the five factors of the PANSS is as follows: positive (4, 28), negative (6, 42), disorganized (3, 21), excited (4, 28), and depressed (3, 21). Individual items of the original PANSS Kay et al. (18) were assigned to these factors based on a consensus five-factor model developed by Wallwork et al. (32).

**Figure 1:**
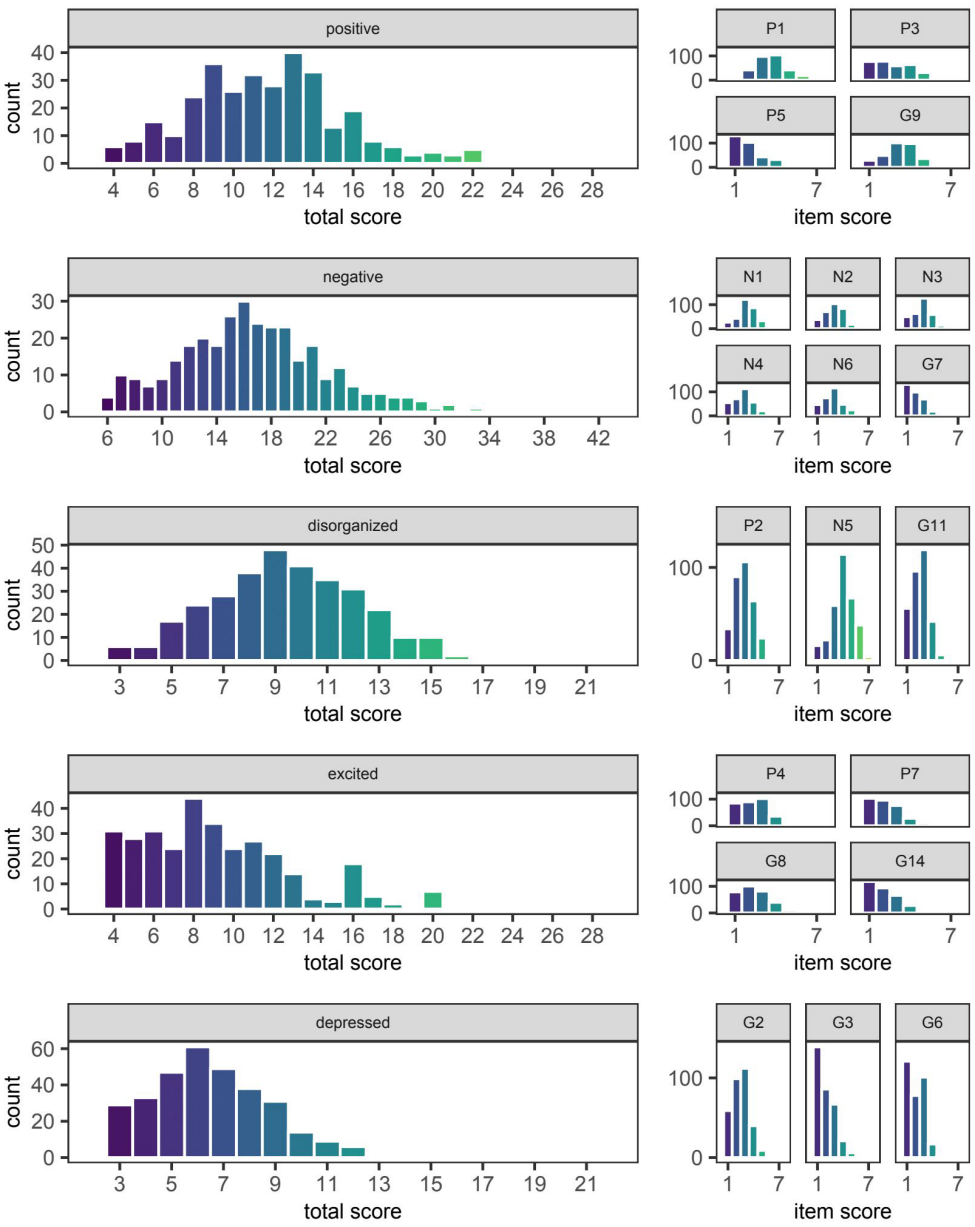
Symptom severity around the time of discharge as quantified by total scores on five different factors of the Positive and Negative Syndrome Scale (PANSS). The median [Q1, Q3] score on the positive, negative, disorganized, excited, and depressed factors of the PANSS was 12 [9, 14], 16 [13, 20], 9 [7, 11], 9 [6, 11], and 6 [5, 8], respectively. The distribution of scores on individual items of the PANSS are shown to the right of the factor to which they were assigned based on the consensus five-factor model developed by Wallwork et al. (32). The naming convention for individual items remains the same as that used by Kay et al. (18) in the original three-factor model (i.e., P1-P7, N1-N7, and G1-G16 denote items numbered 1 through 7 or 1 through 16 of the positive, negative, and general psychopathology scales, respectively).

Histograms depicting the total score on each factor of the PANSS around the time of discharge are presented alongside histograms depicting individual scores on their constituent items in Figure 1. Relative to the median total scores serving as approximations of symptom severity around the time of admission (Table 1), the corresponding values around the time of discharge were unanimously lower (i.e., less severe) across all five factors. In fact, there were: 6 individuals (1.9%) without items assigned to the positive factor (i.e., delusions [P1], hallucinatory behavior [P3], grandiosity [P5], and unusual thought content [G9]); 4 (1.3%) without items assigned to the negative factor (i.e., blunted affect [N1], emotional withdrawal [N2], poor rapport [N3], passive-apathetic social withdrawal [N4], lack of spontaneity and flow of conversation [N6], and motor retardation [G7]); 6 (1.9%) without items assigned to the disorganized factor (i.e., conceptual disorganization [P2], difficulty in abstract thinking [N5], and poor attention [G11]); 31 (9.7%) without items assigned to the excited factor (i.e., excitement [P4], hostility [P7], uncooperativeness [G8], and poor impulse control [G14]); 39 (9.1%) without items assigned to the depressed factor (i.e., anxiety [G2], guilt feelings [G3], and depression [G6]).

### Model evaluation

**Figure 2:**
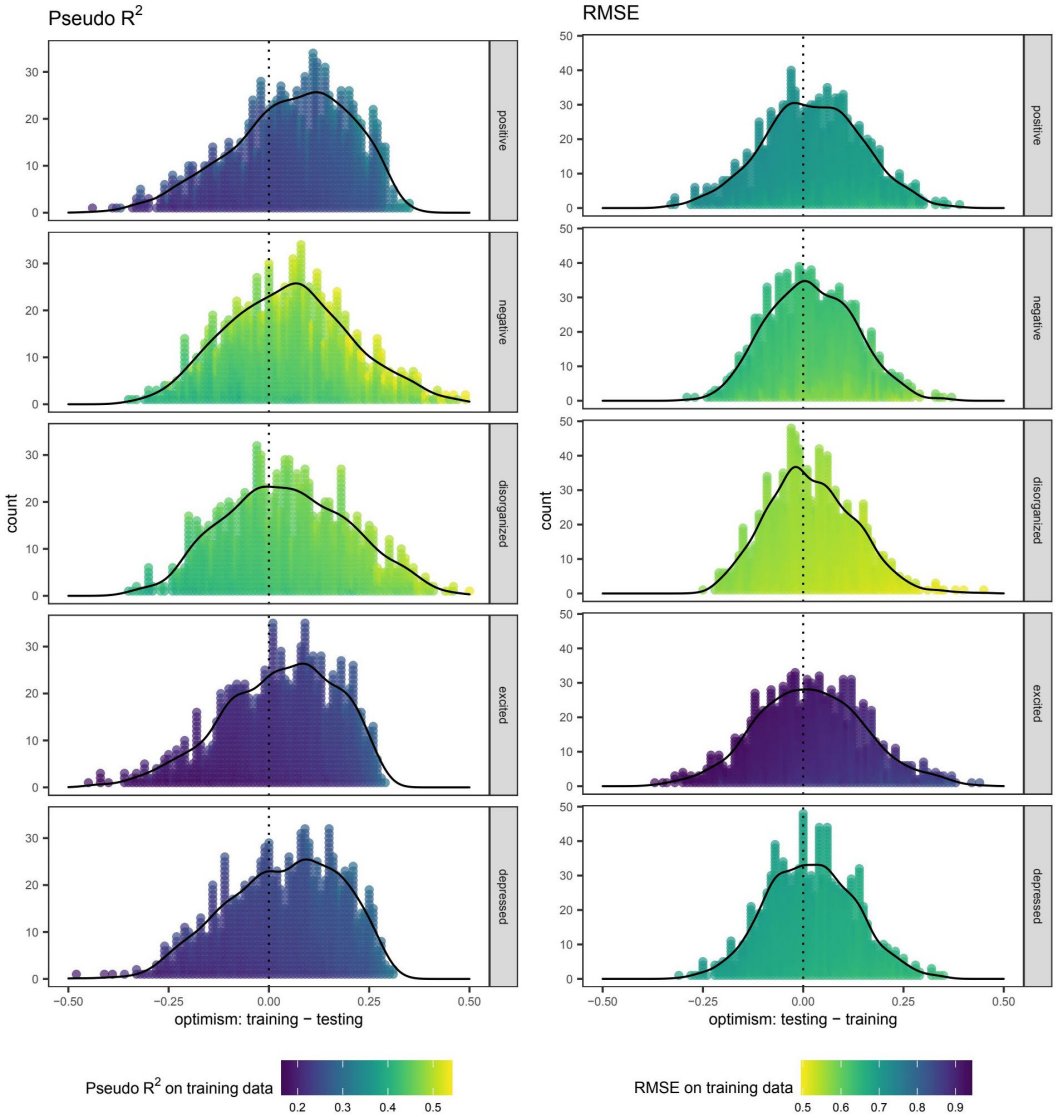
Optimism in the performance of boosted beta regression in predicting symptom severity around the time of discharge as quantified by total scores on five different factors of the Positive and Negative Syndrome Scale (PANSS). Internal validation was accomplished by nested cross-validation. Each point depicts the difference (i.e., optimism) in pseudo *R*^2^ or root mean squared error (RMSE) when using the same boosted beta regression model trained in the outer loop to make predictions on the training and testing data sets. Outcomes were rescaled such that the ensuing values were constrained to the interval (0,1) and approximated the proportion of the maximum attainable score on a given factor of the PANSS. RMSE was therefore on the scale of this rescaled and logit-transformed outcome.

As expected, the pseudo *R*^2^ for each model trained on the full data set was better approximated by the cross-validated value on training data than that on testing data (Table 2). Specifically, the average optimism in pseudo *R*^2^ for models predicting the total score on the positive, negative, disorganized, excited, and depressed factors was 0.05, 0.05, 0.05, 0.03, and 0.04, respectively. Optimism was likewise observed in terms of RMSE with each factor having an average difference between cross-validated values of 0.02 (Table 3). Visualization of this optimism by density curves superimposed on histograms indicates a lesser degree of skewness for RMSE than pseudo *R*^2^ (Figure 2).

**Table 2:** Pseudo *R*^2^ of boosted beta regression models trained to predict symptom severity around the time of discharge as quantified by total scores on five different factors of the Positive and Negative Syndrome Scale (PANSS).

Factor	Pseudo *R*^2^ on all data	Cross-validated pseudo *R*^2^ on training data	Cross-validated pseudo *R*^2^ on testing data
Positive	0.23	0.25	0.20
Negative	0.45	0.46	0.41
Disorganized	0.44	0.45	0.40
Excited	0.21	0.22	0.20
Depressed	0.24	0.24	0.21

Each model was trained to predict the total score on only one of the five factors. Pseudo *R*^2^ was defined as the squared correlation coefficient between observed and predicted values. The first column of values contains in-sample estimates of pseudo *R*^2^ from models trained on the full data set (*N* = 320). The cross-validated estimates in the other two columns represent the mean pseudo *R*^2^ on training or testing data sets partitioned by 100-times 10-fold cross-validation in the outer loop of the nested cross-validation.

**Table 3:** Root mean squared error (RMSE) of boosted beta regression models trained to predict symptom severity around the time of discharge as quantified by total scores on five different factors of the Positive and Negative Syndrome Scale (PANSS).

Factor	RMSE on all data	Cross-validated RMSE on training data	Cross-validated RMSE on testing data
Positive	0.72	0.70	0.72
Negative	0.62	0.62	0.64
Disorganized	0.56	0.55	0.57
Excited	0.90	0.90	0.92
Depressed	0.67	0.67	0.69

Outcomes were rescaled such that the ensuing values were constrained to the interval (0,1) and approximated the proportion of the maximum attainable score on a given factor of the PANSS. RMSE was therefore on the scale of this rescaled and logit-transformed outcome. The first column of values contains in-sample estimates of RMSE from models trained on the full data set (*N* = 320). The cross-validated estimates in the other two columns represent the mean RMSE on training or testing data sets partitioned by 100-times 10-fold cross-validation in the outer loop of the nested cross-validation.

**Figure 3:**
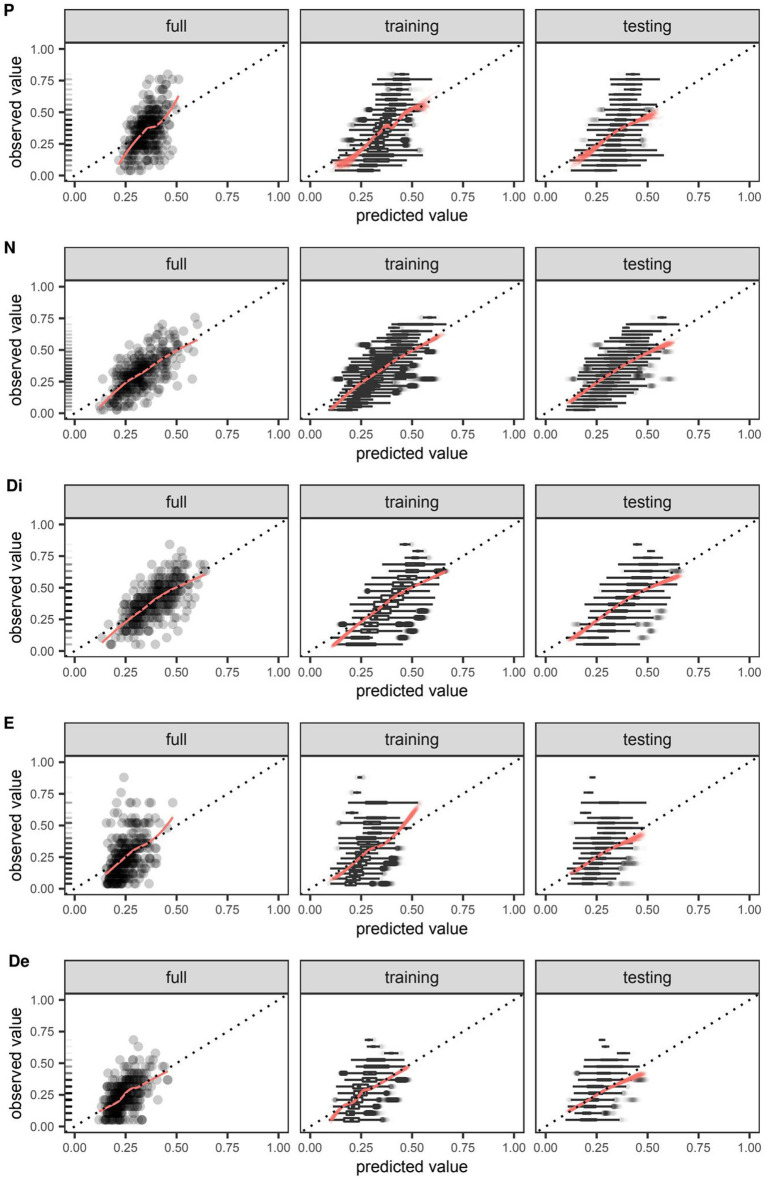
Agreement between observed and predicted values of symptom severity around the time of discharge as quantified by total scores on five different factors of the Positive and Negative Syndrome Scale (PANSS) and predicted by boosted beta regression. Outcomes were rescaled such that the ensuing values were constrained to the interval (0,1) and approximated the proportion of the maximum attainable score on a given factor of the PANSS. For models trained on the full data set and those trained in the outer loop of the nested cross-validation, the extent of agreement between observed and predicted values on the positive (P), negative (N), disorganized (Di), excited (E), and depressed (De) factors were visualized using smooth red curves fitted by locally weighted least squares regression and compared against the dotted identity line. Out-of-sample calibration was uniquely assessed in models trained on the outer loop using the testing data set. Boxplots of observed values were plotted to enhance visualization in cases where it was not feasible to plot individual data points. Rug plots added to the leftmost figure in each row depict the relative frequency of each observed value in the full data set.

Scatter plots depicting the extent of agreement between observations and predictions are presented in Figure 3. Among models trained on the full data set, deviation of the smooth curve from the identity line was most notable for the positive and excited factors. Though this was also reproduced in the cross-validated predictions of training data, the severity of the deviation appeared to be of lesser consequence when examining cross-validated predictions of testing data. The interpretation of PIT histograms derived from predictions on individual testing data sets of the nested cross-validation was limited by the small sample size (*N* = 28), but pooling of these predictions from each repeat in the outer loop yielded histograms that, if noticeably deviated from a uniform distribution, were most concerning for over-forecasting as suggested by low counts in the highest bins. This risk of over-forecasting at the highest bin is consistent with the predilection for overestimation seen in the previous set of scatter plots for cross-validated predictions on testing data (Figure 3).

### Exploratory analysis

**Figure 4:**
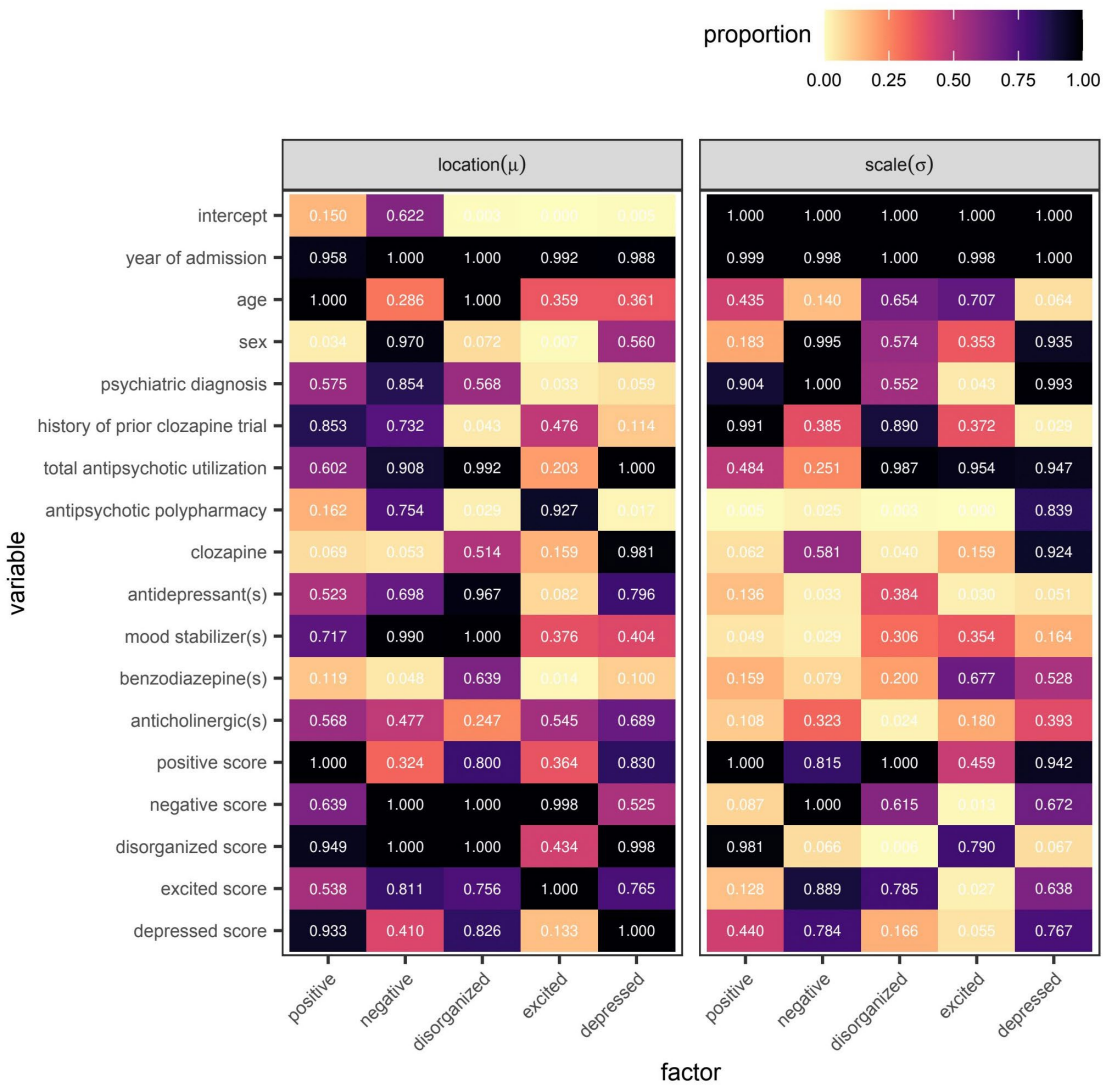
Variable selection frequency within the outer loop of the nested cross-validation for boosted beta regression models predicting symptom severity around the time of discharge as quantified by total scores on five different factors of the Positive and Negative Syndrome Scale (PANSS). Variable selection frequency was defined as the proportion of models re-fitted in the outer loop of the nested cross-validation in which the base learner or base learners specified for a given variable were used to update the additive predictor of interest at least once before termination of the algorithm.

The frequency at which individual variables were selected by the algorithm spanned the range from 0–100% of models trained in the outer loop of the nested cross-validation (Figure 4). No variable was as ubiquitous in its inclusion across all five factors than the year of admission, which had at least one of its specified base learners update 95.8–100% and 99.8–100% of additive predictors for the location and scale parameters, respectively. That said, the total scores around the time of admission were invariably selected when predicting the location parameter of their respective scores around the time of discharge. Total scores on the negative and disorganized factors were also invariably selected when predicting the location parameter of another score around the time of discharge, specifically those of the disorganized and negative factors, respectively.

**Figure 5:**
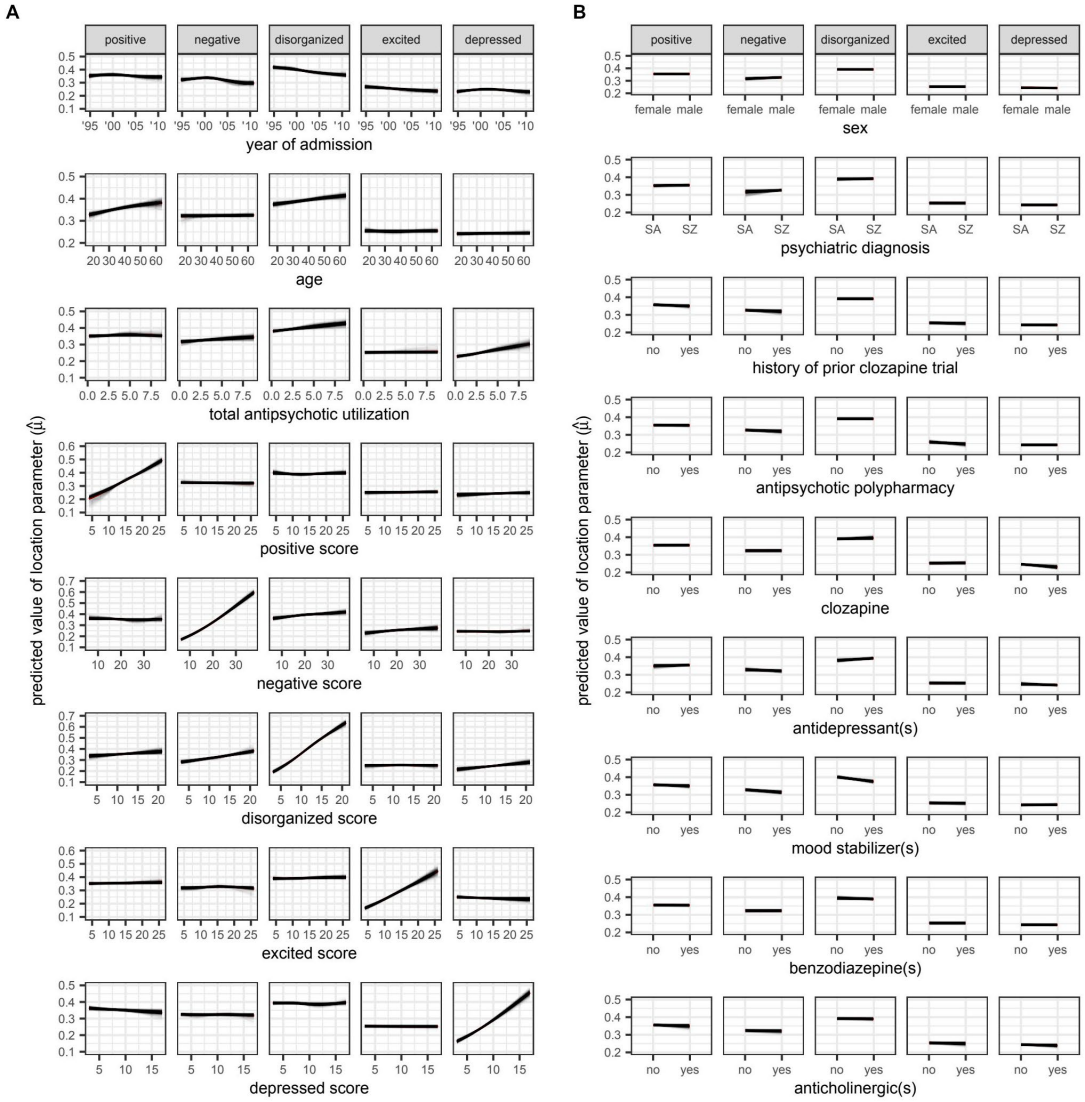
Plots for **A**) numeric and **B**) variables, respectively. SA: schizoaffective disorder. SZ: schizophrenia. Partial dependence plots for candidate predictors of the location parameter [ (μ) ] of beta distributions reflecting symptom severity around the time of discharge as quantified by total scores on five different factors of the Positive and Negative Syndrome Scale (PANSS). Outcomes were rescaled such that the ensuing values were constrained to the interval (0,1) and approximated the proportion of the maximum attainable score on a given factor of the PANSS. Locally weighted least squares regression was used to fit smooth curves for each model trained in the outer loop of the nested cross-validation. The red curve represents the average predicted value across all models at a given value of the candidate predictor. The location parameter is equal to the expected value of the beta distribution (i.e., E(X)=μ ).

**Figure 6:**
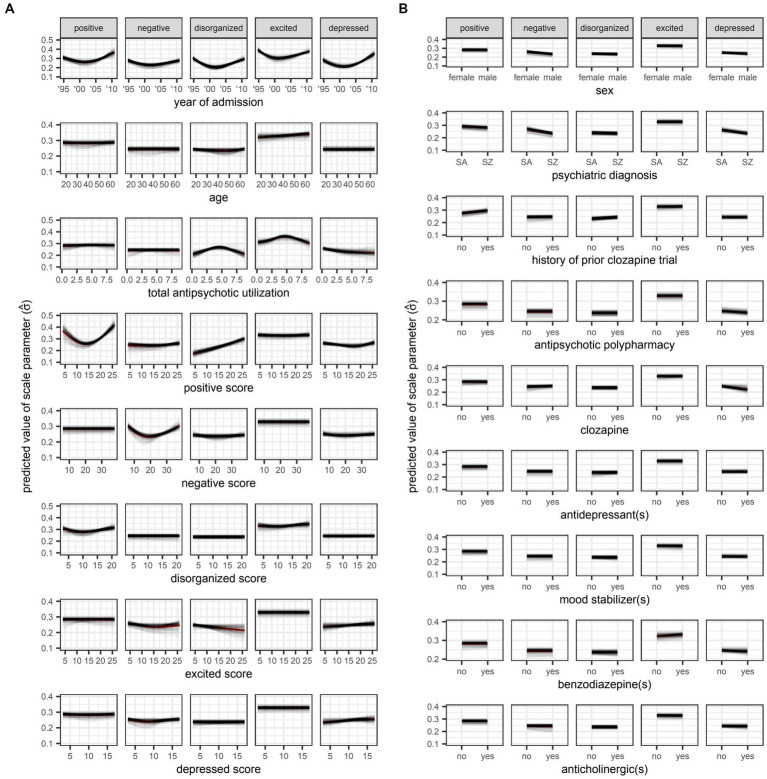
Plots for **A**) numeric and **B**) variables, respectively. SA: schizoaffective disorder. SZ: schizophrenia. Partial dependence plots for candidate predictors of the scale parameter [ (σ) ] of beta distributions reflecting symptom severity around the time of discharge as quantified by total scores on five different factors of the Positive and Negative Syndrome Scale (PANSS). Outcomes were rescaled such that the ensuing values were constrained to the interval (0,1) and approximated the proportion of the maximum attainable score on a given factor of the PANSS. Locally weighted least squares regression was used to fit smooth curves for each model trained in the outer loop of the nested cross-validation. The red curve represents the average predicted value across all models at a given value of the candidate predictor. The square of the scale parameter is proportional to the variance of the beta distribution (i.e., $\mathrm{Var}\left(X\right)=\mu \left(1-\mu \right)\sigma^{2}$ ).

**Figure 7:**
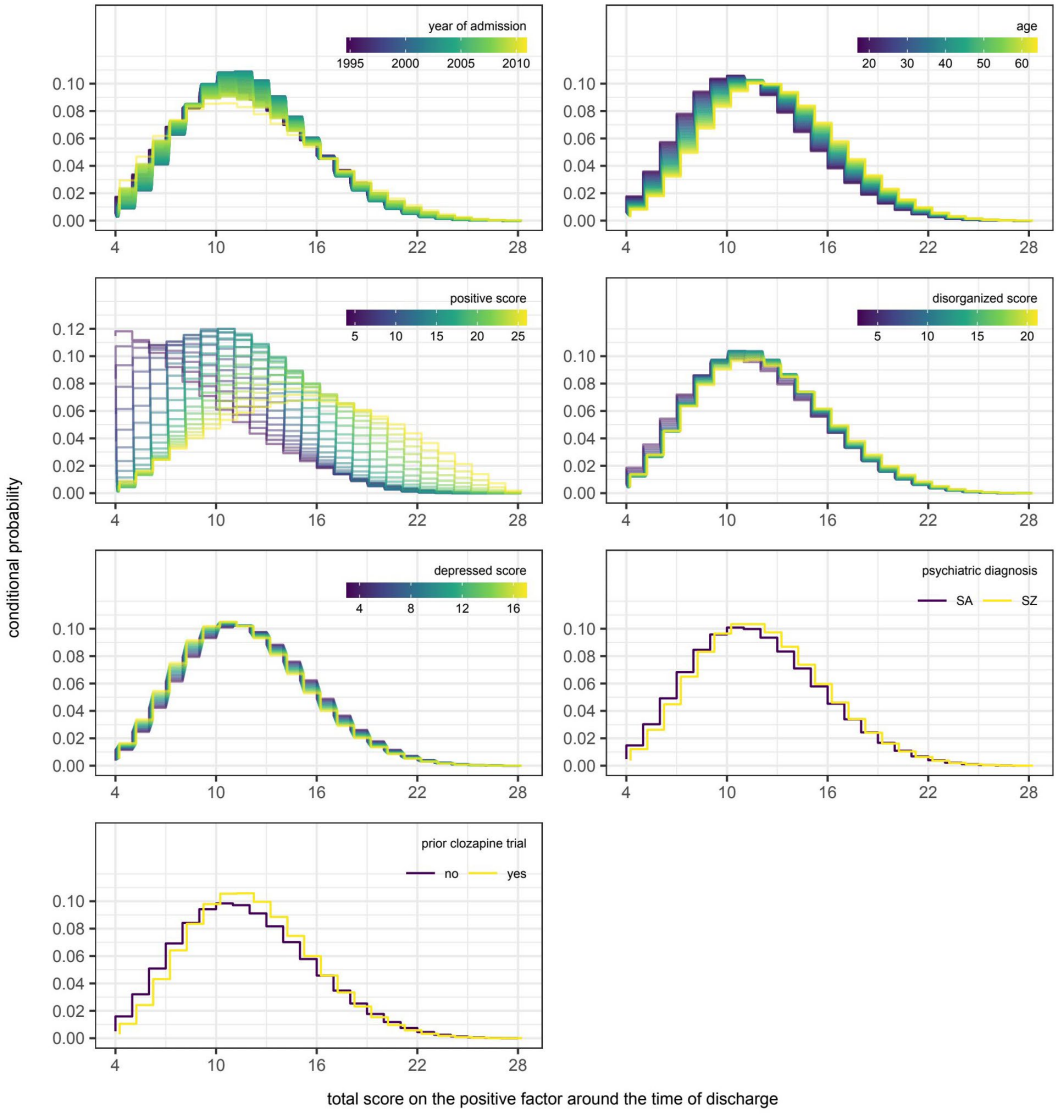
Partial dependence plots for variables included in the boosted beta regression model predicting the total score on the positive factor of the Positive and Negative Syndrome Scale (PANSS) around the time of discharge. The boosted beta regression model was trained on all available data (*N* = 320). The plotting of conditional probabilities required discretization of the predicted beta distributions. Individual items of the PANSS assigned to the positive factor include delusions (P1), hallucinatory behavior (P3), grandiosity (P5), and unusual thought content (G9). The minimum and maximum theoretical scores were therefore 4 and 28, respectively.

**Figure 8:**
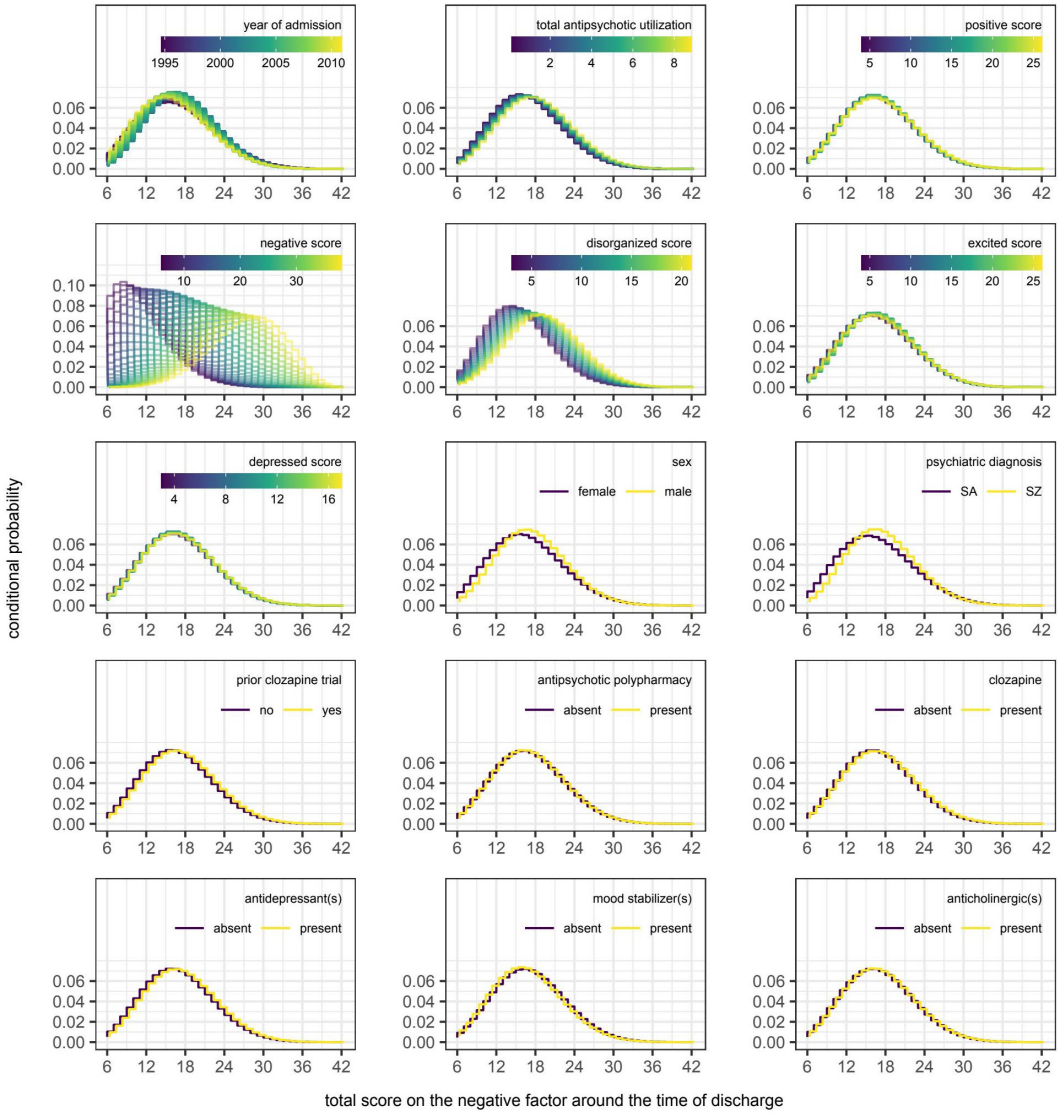
Partial dependence plots for variables included in the boosted beta regression model predicting the total score on the negative factor of the Positive and Negative Syndrome Scale (PANSS) around the time of discharge. The boosted beta regression model was trained on all available data (*N* = 320). The plotting of conditional probabilities required discretization of the predicted beta distributions. Individual items of the PANSS assigned to the negative factor include blunted affect (N1), emotional withdrawal (N2), poor rapport (N3), passive-apathetic social withdrawal (N4), lack of spontaneity and flow of conversation (N6), and motor retardation (G7). The minimum and maximum theoretical scores were therefore 6 and 42, respectively.

**Figure 9:**
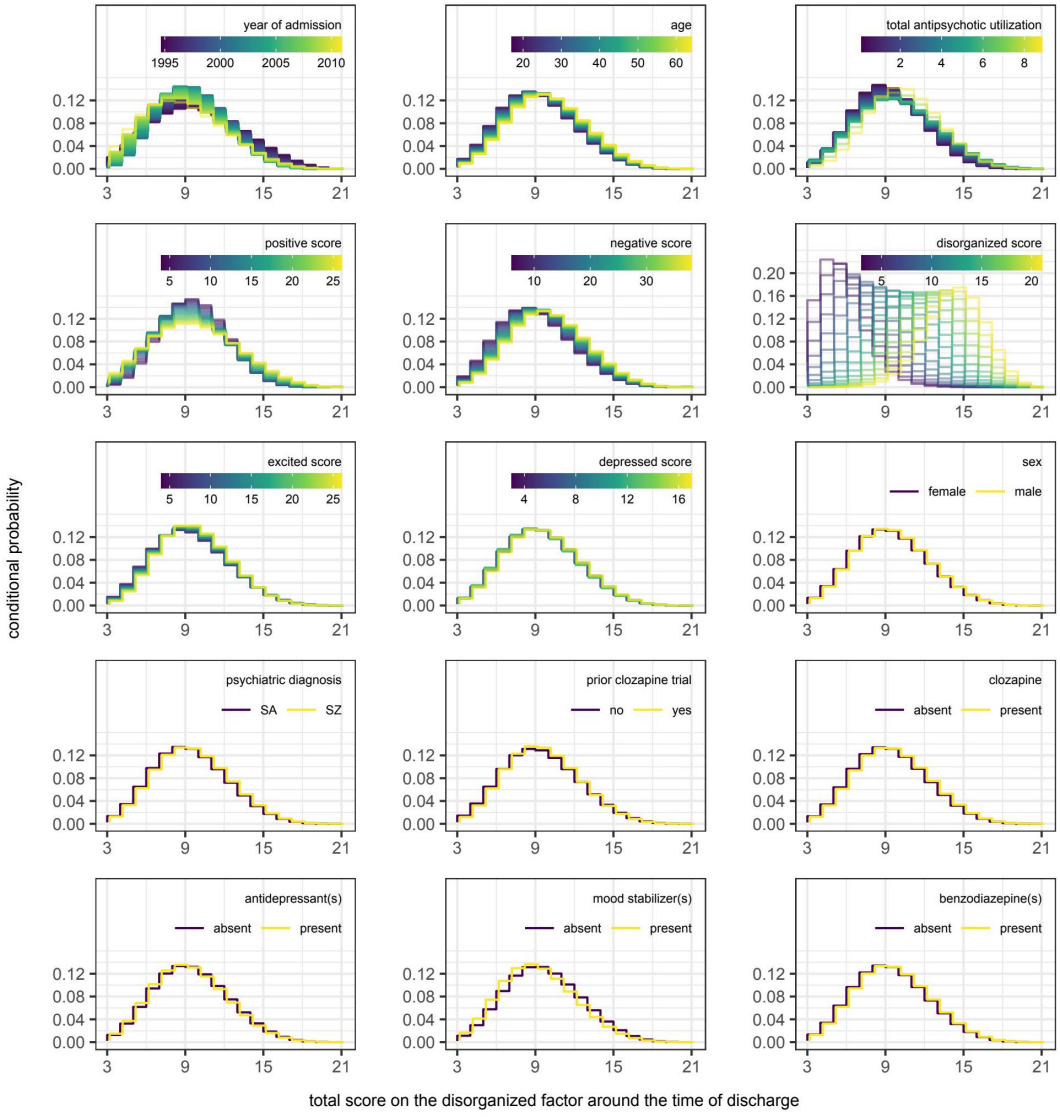
Partial dependence plots for variables included in the boosted beta regression model predicting the total score on the disorganized factor of the Positive and Negative Syndrome Scale (PANSS) around the time of discharge. The boosted beta regression model was trained on all available data (*N* = 320). The plotting of conditional probabilities required discretization of the predicted beta distributions. Individual items of the PANSS assigned to the disorganized factor include conceptual disorganization (P2), difficulty in abstract thinking (N5), and poor attention (G11). The minimum and maximum theoretical scores were therefore 3 and 21, respectively.

**Figure 10:**
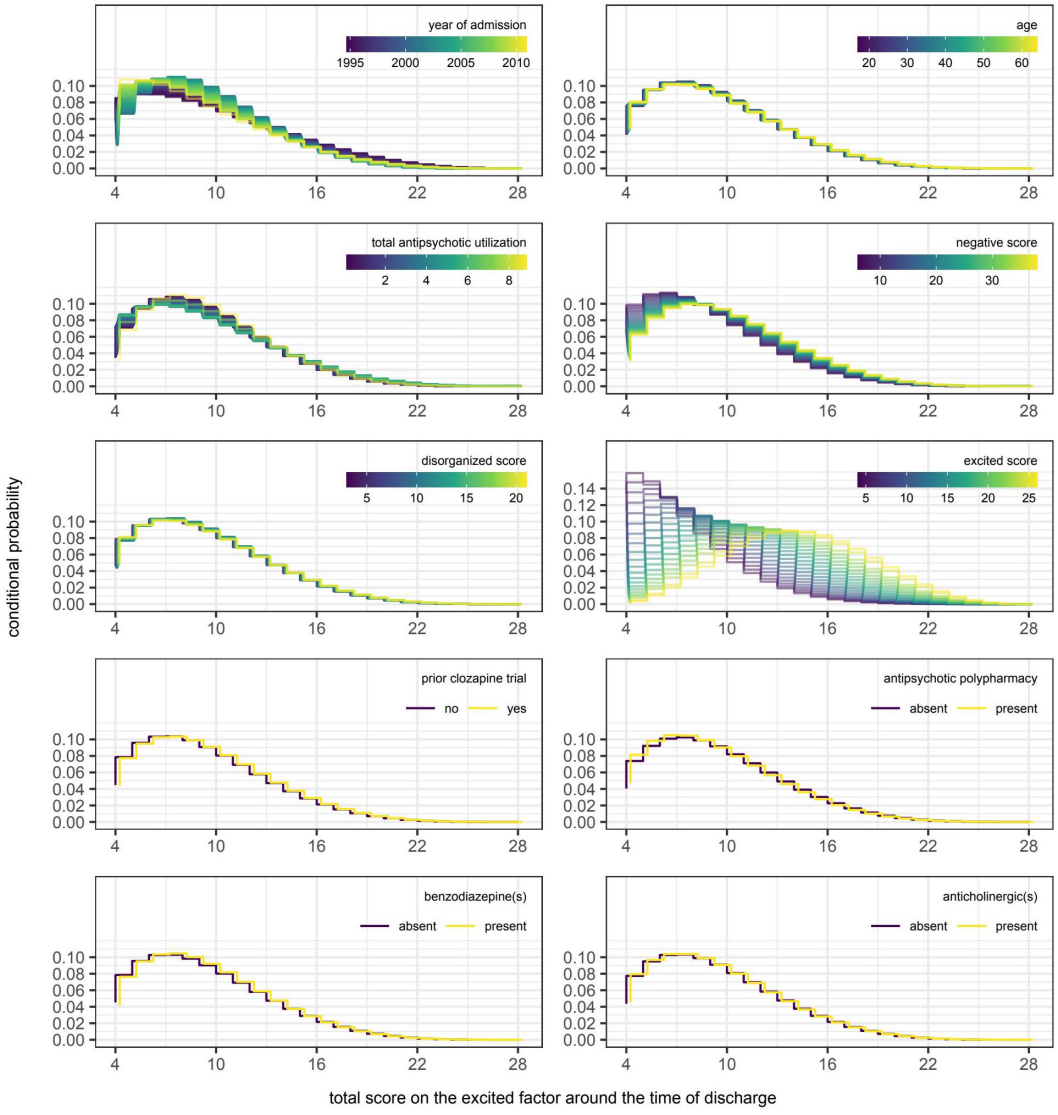
Partial dependence plots for variables included in the boosted beta regression model predicting the total score on the excited factor of the Positive and Negative Syndrome Scale (PANSS) around the time of discharge. The boosted beta regression model was trained on all available data (*N* = 320). The plotting of conditional probabilities required discretization of the predicted beta distributions. Individual items of the PANSS assigned to the excited factor include excitement (P4), hostility (P7), uncooperativeness (G8), and poor impulse control (G14). The minimum and maximum theoretical scores were therefore 4 and 28, respectively.

**Figure 11:**
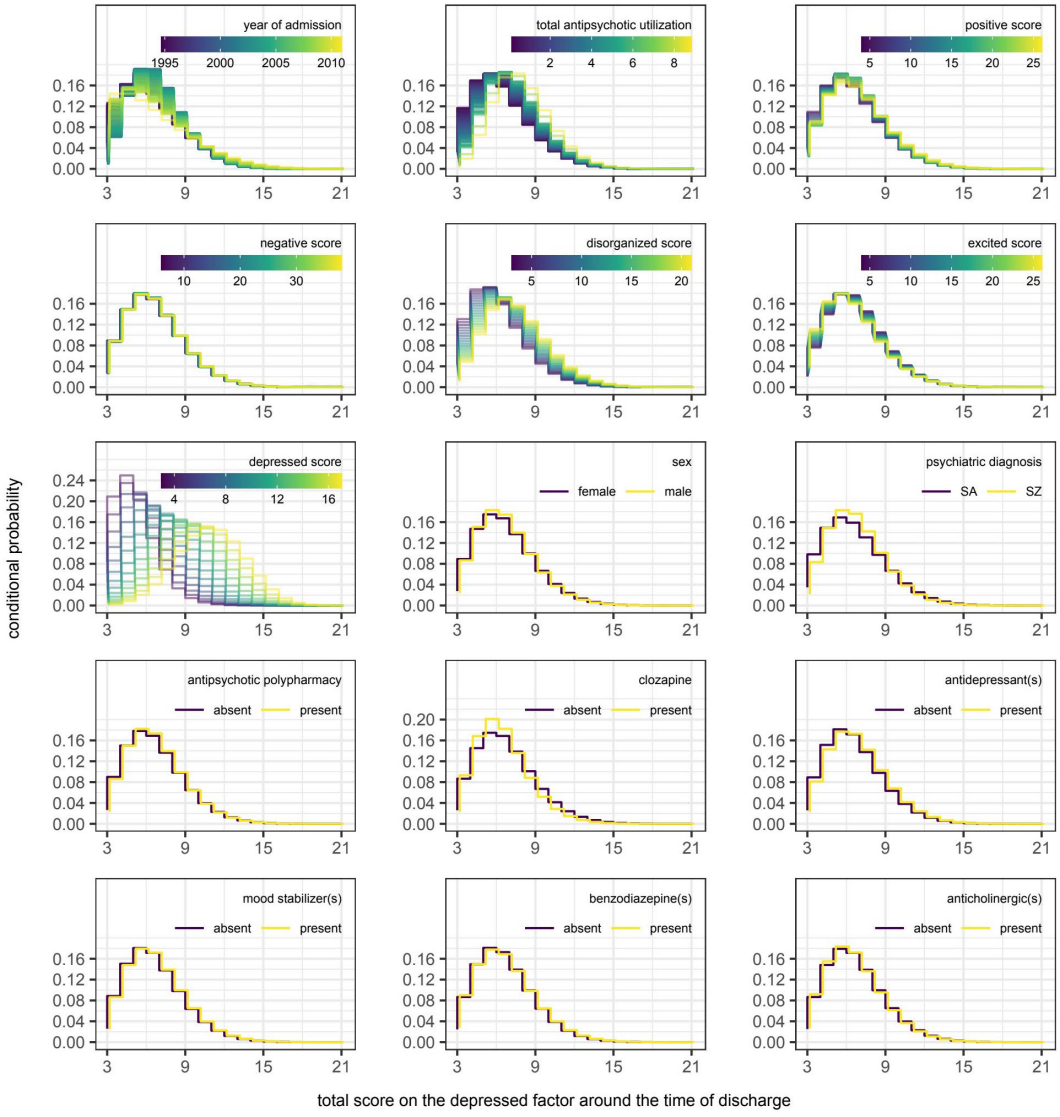
Partial dependence plots for variables included in the boosted beta regression model predicting the total score on the depressed factor of the Positive and Negative Syndrome Scale (PANSS) around the time of discharge. The boosted beta regression model was trained on all available data (*N* = 320). The plotting of conditional probabilities required discretization of the predicted beta distributions. Individual items of the PANSS assigned to the depressed factor include anxiety (G2), guilt feelings (G3), and depression (G6). The minimum and maximum theoretical scores were therefore 3 and 21, respectively.

However, even if a variable were to be regularly selected by the algorithm between runs and repeats of the outer loop of the nested cross-validation, the marginal effect of that variable may be inconsequential if it was used only sparingly to update the additive predictor. As illustrated in Figures 5, 6, the difference in estimates of a distribution parameter between two values of any binary variable is typically smaller than that between two values of any numeric variable taken over the range of observations. These discrepancies in marginal effect are also apparent in the partial dependence plots generated from models trained on the full data sets following the discretization of predicted beta distributions (Figures 7–11). The fitted probability mass functions tended to be most varied in form when adjusting the value of numeric predictors. Beyond the sizeable effect that previous scores have on the prediction of future scores, the effect that disorganized symptoms around the time of admission have on negative symptoms around the time of discharge is also readily appreciable on inspection of the partial dependence plots and straightforward in its interpretation as it has a comparatively larger effect on the location parameter than the scale parameter.

## Discussion

Expectations for the performance of any prediction model should consider potential limitations inherent to the data set. First, the current data used to predict psychosis at discharge are taken from a relatively short period in time taken weeks or months in advance of the discharge date. For that reason, any semblance of an early clinical response to clozapine suggestive of a more favorable trajectory (47) would have gone undetected because there is no follow-up assessment, save for the one around discharge, with which to compare. Second, the self-imposed restriction to use only reliable and readily available information found in most medical records means that the scope of the data is largely limited to routine assessments; more complex potential predictors, such as MRI brain scans, biomarkers or genetic data as might be gathered in a research study were not available. Thus, this restriction, though a potential hindrance to model performance, was deliberately introduced in hopes of maximizing the utility of these models in the setting of routine clinical practice. Third, individuals responsible for scoring the PANSS were not blinded to the treatments received at the program. Inter-rater reliability was also not assessed in this study. That said, the performance of each rater was reviewed annually using standardized interviews to ensure that ratings on individual items were all within one point of the standard score (19).

Efforts were made to adhere to the transparent reporting of a multivariable prediction model for individual prognosis or diagnosis (48), but the decision to use boosted beta regression precluded conventional estimation of confidence intervals and *p*-values. Though it has been established that cross-validated estimates on testing data reflect the expected performance of the model fitting procedure on hypothetical data sets sampled from a distribution identical to that of the observations, work aimed at quantifying the confidence interval of these estimates is still in progress and has yet to be extended beyond linear models (49). Even so, the degree of optimism associated with boosted beta regression as depicted in Figure 2 can be helpful in visualizing the spread of values around cross-validated estimates of pseudo *R*^2^ and RMSE because the corresponding estimates on training data were largely consistent between runs.

Insofar as it is possible to compare performance across different outcomes, it would appear that the negative and disorganized factors of the PANSS are more amenable to prediction using data that were readily available around the time of admission. Heterogeneity in treatment response may explain the comparatively poor performance on the positive factor. While the latent variable corresponding to positive symptoms has previously been shown to be the most improved following treatment at this tertiary program (19), it is important not to conflate the response of the group with that of the individual. Owing to the clinical phenomenon that is ultra-treatment-resistant psychosis, clozapine may prove beneficial in the majority of patients yet still be unable to alleviate symptoms to an acceptable degree in approximately 40% of those meeting the criteria for treatment-resistant schizophrenia (50–53). Unless the models are trained using data capable of distinguishing clozapine responders from non-responders, the ability to predict total scores on the positive factor will be limited given the integral role of this antipsychotic at the tertiary program (17), despite its multitude of side-effects (54–57). Unfortunately, differences between these two groups have been elusive. It is unlikely that this situation would be much improved had biomarkers been available as much of the research is in its infancy (58).

That said, two clinicodemographic characteristics were associated with a sustained response to clozapine monotherapy in a retrospective chart review of patients who had previously taken or were currently taking this antipsychotic for treatment-resistant schizophrenia or schizoaffective disorder: (1) a shorter delay to the initiation of treatment and (2) fewer hospitalizations over that same period of time (59). This first association appears to be particularly well established since the same research group had previously found longer delays to be associated with poorer outcomes in a systematic review of four other retrospective studies on the subject (60). Unfortunately, neither characteristic was readily available for this analysis because they were either missing or recalled with questionable reliability with no alternate means for verification. Even if they were available, there is no guarantee that their inclusion would have greatly improved the performance of the models because a majority of patients (58.1%) had already tried clozapine before their admission. Considering the reason for their referral, it is likely that a sizeable proportion of this majority would have already met the criteria for ultra-resistant-treatment psychosis at admission if they had otherwise tolerated this antipsychotic well in the past. Having a prior history of clozapine use, let alone a history of non-response, has been grounds for exclusion from studies examining potential predictors of response to clozapine monotherapy. The overall lack of research involving this patient population also explains the continued reliance on expert consensus when the augmentation of clozapine monotherapy is necessary (61).

Interestingly, of the 119 patients in the aforementioned chart review who were retrospectively classified as clozapine non-responders at the first time point (i.e., during an assessment made within the first 2 years of treatment initiation), 15 (12.6%) were classified as clozapine responders at the second time point (i.e., during the most recent assessment in which clozapine was still prescribed to the patient) (59). Conversely, of the 122 patients classified as clozapine responders at the first time point, 20 (16.4%) were classified as clozapine non-responders at the second time point. Together, these two trajectories give the impression that ultra-treatment resistance is potentially reversible, but may also present later in individuals currently responding to clozapine monotherapy. Thus, while it may appear reasonable to expect individuals with ultra-treatment-resistant psychosis at admission to require treatment augmentation at discharge and hence have a higher symptom burden relative to those adequately treated with clozapine alone, this is unlikely to be true in all cases. Limited sample sizes prevented meaningful assessment of the differences that may exist between stable non-responders and converted responders. Again, further research in this area is required to improve prognostication.

Should the above explanation for the comparatively poor performance of the model predicting the total score on the positive factor be correct, it then raises the question of whether this heterogeneity in treatment response also extends to the excited and depressed factors. The therapeutic benefits of antipsychotics may not be restricted to the improvement of positive symptoms as has been previously speculated. Based on the results of a post-hoc analysis, improvement on a general second-order factor may underlie improvement on each of the five factors of the PANSS in patients not previously known to have either treatment-resistant or ultra-treatment-resistant psychosis (62). If this hierarchical structure of symptom improvement is also applicable in the setting of treatment-resistant and ultra-treatment-resistant psychosis, then it stands to reason that the prediction of total scores on the other four factors could be similarly impacted. To our knowledge, the generalizability of this finding to the patient population of interest has yet to be formally tested. Furthermore, an additional explanation would be needed to reconcile the apparent difficulty in predicting treatment response with the seemingly better predictions obtained on the negative and disorganized factors of the PANSS. Perhaps response to clozapine can be predicted to some extent given the inclusion of age and baseline negative symptom severity, albeit on the five-factor rather than the three-factor PANSS (63), but the positive, excited, and depressed factors load less strongly on the general second-order factor, resulting in a weaker correlation between the first-and second-order latent variables.

The decision to predict the total scores on each of the five factors of the PANSS is uncommon in that models are more frequently trained to predict response in refractory psychosis. However, a 20% reduction in symptom severity from baseline values may not even begin to reflect the smallest increment of change that can be routinely detected by clinicians (64). In addition, a focus on this threshold discourages a more thorough investigation into the underlying dimensions of psychotic disorders. Should the prediction of individual symptoms become even more important given the growing interest in applying network analysis to mental disorders (65), the current approach could be used to predict scores on individual items instead.

Lastly, it is worth reiterating that the primary objective of this study was to train models for prediction rather than inference. For that reason, caution must be exercised when interpreting the variables selected by the non-cyclic algorithm. Variables may be inconsistently selected within the refitted models of the nested cross-validation because of algorithmic instability or the presence of mutually correlated variables (66, 67). Stated differently, the relationships between predictors and the outcome reflect association do not imply causality. Thus, the goal was never to create models to guide decision-making, but to identify individuals likely to have more severe symptoms around the time of discharge, allowing for an earlier and more thorough assessment of their needs at the earliest time point possible.

In summary, patient-level responses to personalized treatments offered at a tertiary-care program for treatment-resistant psychosis were approximated by the total scores on individual factors of the PANSS around the time of discharge. These values were predicted using a novel approach that better lends itself to the modelling of integer scores from Likert scales commonly encountered in psychiatry. Safeguards in the form of data resampling methods were put in place to mitigate the risk of overfitting the models to the noise in the training data. This proved successful since the average optimism in predictive ability was kept to a minimum when comparing the performance on data that were used to train each model to that on data held out from this procedure. Candidate predictors derived from clinicodemographic information and prescription drug data were of varying and occasionally inconsistent prognostic value. That said, symptom severity around the time of admission proved most influential in estimating the corresponding value around the time of discharge.

Few investigations into the prognostication of outcomes in treatment-resistant psychosis exist, let alone those employing techniques traditionally associated with machine learning (68). Thus, ongoing work is required should precision medicine ever have a hope of becoming the standard of practice in psychiatry. Progress toward this endeavour may be helped by the adoption of statistical tools adapted to the handling Likert scales given the loss of information when converting raw scores into categories for the sole purpose of classification (e.g., responder versus non-responder). The approach detailed here represents one method in which clinical data can be used to their fullest potential.

Another key point to be emphasized from this investigation is the importance of including the PANSS in routine clinical practice – especially for complex patients maintained in a tertiary care, inpatient setting. There are multiple reasons for the clinical underutilization of this “gold standard” comprehensive measure of psychosis, which – as noted above – assesses multiple different and independent domains. Opler and colleagues (69) note that the PANSS, compared to other rating scales, has “many items, evaluates a multidimensional array of symptoms … and involves the use of data from patient reports, caregiver reports, and clinical observations. Consequently, the PANSS takes up more time during training and requires a greater amount of time for one to master it compared to many other instruments.” Similarly, Østergaard and colleagues (70) emphasize the time required to complete the PANSS, as well as challenges administering the scale to severely ill patients. Nevertheless, the comprehensive profile of psychosis in patients provided by the PANSS would suggest that it should be used more frequently in clinical practice, which may require programs to implement specialized training for the scale (69) or consider using an abbreviated version, such as the PANSS-6 (70).

### Conclusion

Boosted beta regression—selected for its ease in modelling non-linear effects with P-spline base learners as well as its ability to perform shrinkage estimation and variable selection—showed promise in predicting symptom severity around the time of discharge from a tertiary care program for treatment-resistant psychosis. Correlation between predictions and observations were highest for the negative and disorganized factors of the PANSS when using only reasonable reliable and readily available information around the time of admission. Future studies may benefit from a richer training data set with even more clinicodemographic variables.

## Data availability statement

The datasets presented in this article are not readily available because Data underlying the study are not available due to restrictions imposed by the University of British Colombia Clinical Research Ethics Board. Data are stored at the British Columbia Psychosis Program (BCPP) in Vancouver, Canada, which is an inpatient medical unit of the BC Provincial Health Services Authority (PHSA). Data are available to PHSA employees who have had required background checks, as well as affiliated members of the BCPP, pending addition to the study protocol. As the data are not fully anonymized, total free access to data by all is not permitted. Requests to access the datasets should be directed to al.barr@ubc.ca

## Ethics statement

The studies involving human participants were reviewed and approved by the University of British Columbia. Written informed consent for participation was not required for this study in accordance with the national legislation and the institutional requirements.

## Author contributions

LL was responsible for data collection, statistical analysis, and writing the first draft of the manuscript. AB was responsible for study design, REB approval, and supervision. WH was responsible for securing project funds. All authors contributed to the article and approved the submitted version.

## Funding

The research was supported by an NSERC grant to AB and an unrestricted grant through the British Columbia Provincial Health Services Authority. The funders had no role in study design, data collection and analysis, decision to publish, or preparation of the manuscript.

## Conflict of interest

RP reports personal fees from Janssen, Lundbeck and Otsuka. WH reports personal fees from Canadian Agency for Drugs and Technology in Health, AlphaSights, Guidepoint, Translational Life Sciences, Otsuka, Lundbeck, and Newron; grants from Canadian Institutes of Health Research, BC Mental Health and Addictions Services; and has been a consultant (non-paid) for *In Silico*.

The remaining authors declare that the research was conducted in the absence of any commercial or financial relationships that could be construed as a potential conflict of interest.

## Publisher’s note

All claims expressed in this article are solely those of the authors and do not necessarily represent those of their affiliated organizations, or those of the publisher, the editors and the reviewers. Any product that may be evaluated in this article, or claim that may be made by its manufacturer, is not guaranteed or endorsed by the publisher.
